# The effect of adherence to guidelines for initial antiretroviral therapy on 1-year outcomes: a French cohort study

**DOI:** 10.1186/s12879-014-0596-y

**Published:** 2014-11-14

**Authors:** Laurent Cotte, Thomas Bénet, Philippe Vanhems, Corinne Brochier, Thomas Perpoint, Tristan Ferry, Christian Chidiac

**Affiliations:** Department of Infectious and Tropical Diseases, Hôpital de la Croix-Rousse, Hospices Civils de Lyon, Lyon, France; INSERM, U1052, Lyon, France; Infection Control and Epidemiology Unit, Edouard Herriot Hospital, Hospices Civils de Lyon, Lyon, France; University Claude Bernard - Lyon-1, UMR CNRS, 5558 Lyon, France; Clinical Research Unit, Hôpital de la Croix-Rousse, Hospices Civils de Lyon, Lyon, France; University Claude Bernard - Lyon-1, Lyon, France; CIRI, INSERM U1111, Lyon, France

**Keywords:** HIV infection, Antiretroviral treatment, Adherence to guidelines, Prognosis

## Abstract

**Background:**

Guidelines for antiretroviral treatment (cART) are published regularly, but there is little information regarding the effect of adherence to guidelines on patient outcomes. We assessed the effect of following the "when-to-start" and "what-to-start" guidelines, on treatment modifications, and on immunological and virological outcome at 12 months in a cohort of HIV-1 infected patients initiating cART from 2000 to 2010.

**Methods:**

Consecutive HIV-1 infected patients, antiretroviral naive, initiating cART from 2000 to 2010 at a University Hospital were enrolled. HIV-2 infection, cART for prevention of mother-to-child transmission or during primary HIV-infection and unlicensed drugs were excluded. The respect or not of the "when-to-start" and "what-to-start" guidelines was based on French guidelines published from 2000 to 2010. Factors associated with cART modifications at 12 months and factors associated with an HIV viral load of <50 copies/mL at 12 months were assessed by univariate and multivariate logistic regression modeling. Variations in CD4 counts from baseline were assessed by univariate and multivariate linear regression.

**Results:**

Of 1365 patients starting cART, 151 were treated outside "when-to-start" guidelines and 150 were treated outside "what-to-start" guidelines. Not using "when-to-start" guidelines was mainly related to early initiation in young men having sex with men, and was not associated with a significantly different outcome at 12 months. Treatments that did not follow "what-to-start" guidelines were not observed in any specific population and were associated with more treatment modifications and a poorer virological outcome at 12 months.

**Conclusions:**

Adherence to "what-to-start" guidelines is associated with a better outcome at 12 months in HIV-infected patients initiating antiretroviral therapy. Efforts should be made to promote adherence to these guidelines.

## Background

The introduction of combined antiretroviral therapy (cART) in 1996 led to a dramatic improvement in HIV disease prognosis, from a deadly disease to a manageable chronic infection compatible with a normal or near-normal life expectancy [1]-[3]. Since then, several national or international scientific societies have published guidelines to help physicians decide the optimal time to start antiretroviral treatment ("when-to-start" guidelines) and the optimal antiretroviral combinations to use ("what-to-start" guidelines) [4]-[7]. Guidelines are updated regularly based on availability of new drugs and evidence from clinical trials. Most national and international consensus guidelines are broadly similar in their recommendations, particularly around "what-to-start".

Following the guidelines depends on the availability of the drugs and the organization of the health-care system, but also on physicians’ personal experience. For example, prescription of not recommended drugs may occur because of the guidelines lag behind clinical trial results but also in specific clinical situations such as HCV or HBV coinfections, renal insufficiency or treatment of opportunistic infections. The availability of numerous drugs facilitates the prescription of various combinations in industrialized countries, resulting in a significant proportion of prescriptions outside the recommendations [8].

Few studies have reported adherence to guidelines in industrialized countries [9]-[15]. Some of these studies included patients who started antiretroviral treatment before the cART era [10] or shortly after [11],[12],[14], a time when suboptimal treatments such as dual-nucleoside combinations were still prescribed. Most of these studies focused on adherence to guidelines and little information was available regarding the effect of adherence to guidelines on the outcome of cART [14].

We conducted an analysis of first line cART in a large cohort of patients who initiated cART from 2000 to 2010. We hypothesized that good adherence to guidelines would improve patients' outcome, independent of the time period. One-year outcomes were studied according to whether or not the patient's treatment followed the French national guidelines applicable during the time of treatment.

## Methods

### Study population and design

The Department of Infectious and Tropical Diseases at Croix-Rousse Hospital in Lyon, France, follows approximately 2,500 HIV-infected patients yearly. The Department provides care to patients from the diagnosis of HIV infection to complications from AIDS, leading to a high retention rate. This hospital cohort is included in the French Hospital Database on HIV and follows the protocols described elsewhere [16]. patients' files are registered in a computerized database after the patient provides written informed consent. The database is registered by the French Commission Nationale de l’Informatique et des Libertés under the reference CNIL 1599796. The study was performed in accordance with The ICHGCP guidelines and the Declaration of Helsinki. Every treatment-naïve adult patient, initiating cART from January 2000 to December 2010, and for whom baseline and 12-month data were available (or deceased before month 12) were included. Patients with HIV-2 infection or who were initiating cART for the prevention of mother-to-child transmission only, those initiating cART during primary HIV infection, or who were using unapproved cART drugs in a clinical trial were excluded.

### Data collection

Demographic and biological data at baseline and at 12 months were recorded. The time from HIV diagnosis to cART initiation was also recorded, as well as the duration of the first line of treatment and the reason for treatment modification or interruption if it occurred during the first year.

### Description of outcomes and risk factors

The timing of cART initiation ("when-to-start" guidelines) was defined as "recommended", "optional", or "not recommended" on the basis of actual CD4 count, HIV viral load, and other specific conditions such as hepatitis B virus (HBV) or hepatitis C virus (HCV) coinfection, high cardio-vascular risk, or treatment as prevention [17]-[22]. Since guidelines were not published at a defined date, 2-years intervals were considered for determining whether guidelines were followed (e.g. 2002 guideline was considered for treatment initiation from January 2002 to December 2003). These 2-years intervals were labeled by the year of publication of the guidelines. The period of time from 2000-2010 was retained for this study for homogeneity of cART, since dual-nucleosides combinations were no longer recommended and boosted protease inhibitors started to be widely used in France beginning in the year 2000. Recommended and optional cART were grouped together for analysis. Table 1a summarizes the French "when-to-start" guidelines from 2000 to 2010.

**Table 1:** **French guidelines for cART initiation from 2000 to 2010**

a "when-to-start" guidelines						
	2000	2002	2004	2006	2008	2010
CDC B-C	Rec.	Rec.	Rec.	Rec.	Rec.	Rec.
CD4 < 200/mm^3^	Rec.	Rec.	Rec.	Rec.	Rec.	Rec.
CD4 < 15%	Rec.	Rec.	Rec.	Rec.	Rec.	Rec.
CD4 200-349/mm^3^	Rec.	Opt. (1)	Rec.	Rec.	Rec.	Rec.
CD4 350-500/mm^3^		NR	Opt. (1)	Opt. (1)	Opt. (2)	Rec.
CD4 > 500/mm^3^		NR	NR	NR	NR	Opt. (3)
**b "what-to-start" guidelines**						
	**2000**	**2002**	**2004**	**2006**	**2008**	**2010**
NRTIs Backbone						
ZDV + ddI	Pref.	Pref.	Alt.	Pref.	NR	NR
ZDV +3TC	Pref.	Pref.	Pref.	Pref.	Alt.	Alt.
d4T + ddI	Pref.	NR	NR	NR	NR	NR
d4T +3TC	Pref.	Pref.	Alt.	NR	NR	NR
ZDV + FTC		Alt.	Pref.	Pref.	Alt.	Alt.
TDF +3TC		Alt.	Pref.	Pref.	Pref.	Alt.
TDF + FTC		Alt.	Pref.	Pref.	Pref.	Pref.
ddI +3TC		Alt.	Pref.	Pref.	Pref.	NR
ddI + FTC		Alt.	Pref.	Pref.	Pref.	NR
ABC +3TC		Alt.	Pref.	Pref.	Pref.	Pref.
ABC + FTC		Alt.	Pref.	Pref.	Pref.	Alt.
ddI + TDF		Alt.	Alt.	NR	NR	NR
3 NRTIs combinations						
ABC +3TC + ddI		Alt.	NR	NR	NR	NR
ABC +3TC + d4T		Alt.	NR	NR	NR	NR
ZDV +3TC + ddI		Alt.	NR	NR	NR	NR
ABC +3TC + TDF		Alt.	NR	NR	NR	NR
TDF + ddI +3TC		Alt.	NR	NR	NR	NR
ZDV +3TC + ABC	Pref.	Pref.	Alt. (1)	Alt. (1)	NR	NR
Other 3 NRTI combinations		Alt.	NR	NR	NR	NR
Unboosted PIs						
RTV	Pref.	NR	NR	NR	NR	NR
IDV	Pref.	NR	NR	NR	NR	NR
NFV	Pref.	Pref.	Alt.	NR		
Boosted PIs						
APVr	Alt.	Alt.				
SQVr	Pref.	Pref.	Pref.	Pref.	Alt.	Alt.
IDVr	Alt.	Pref.	Pref.	Alt.	Alt.	Alt.
LPVr	Alt.	Pref.	Pref.	Pref.	Pref.	Pref.
fAPVr			Pref.	Pref.	Pref.	Alt.
ATVr				Alt.	Pref.	Pref.
DRVr				NR	Alt.	Pref.
NNRTIs						
EFV	Pref.	Pref.	Pref.	Pref.	Pref.	Pref.
NVP	Pref.	Pref.	Pref.	Alt.	Alt.	Alt.
Integrase Inhibitor						
RAL						Alt.
Antiretroviral combinations						
NNRTI + PI (boosted or not)				NR	NR	NR
2 NRTIs +1 NNRTI	Pref.	Pref.	Pref.	Pref.	Pref.	Pref.
2 NRTIs +1 PI	Pref.	Pref.	Alt.	NR	NR	NR
2 NRTIs +1 boosted PI	Pref.	Pref.	Pref.	Pref.	Pref.	Pref.
Boosted PI monotherapy				NR	NR	NR

*Abbreviations*: *Rec* ART recommended, *Opt* ART optional, NR ART Not recommended; (1), if HIV-RNA >10^5^ log copies/ml; (2), if HIV-RNA >10^5^ log copies/ml >50 years, HBV/HCV coinfection or high cardiovascular risk; (3), if HIV-RNA >10^5^ log copies/ml, >50 years, HBV/HCV coinfection, high cardiovascular risk or treatment as prevention.

*Abbreviations*: *ZDV* zidovudine, *ddI* didanosine, *3TC* lamivudine, *d4T* stavudine, *FTC* emtricitabine, *TDF* tenofovir, *ABC* abacavir, *NRTI* nucleoside/nucleotide reverse transcriptase inhibitors, *PI* protease inhibitor, *RTV* ritonavir, *IDV* indinavir, *NFV* nelfinavir, *APVr* boosted amprenavir, *SQVr* boosted saquinavir, *IDVr* boosted indinavir, *LPVr* boosted lopinavir, *fAPVr* boosted fosamprenavir, *ATVr* boosted atazanavir, *DRVr* boosted darunavir, *NNRTI* non-nucleoside reverse transcriptase inhibitors, *EFV* efavirenz, *NVP* nevirapine, *RAL* raltegravir, Pref preferred, *Alt* alternative, *NR* Not recommended; (1), if HIV-RNA <10^5^ log copies/ml.

The choice of antiretrovirals and the type of combination ("what-to-start" guidelines) was defined as "preferred", "alternative", or "not recommended". Since new drugs were often classified as "alternative" before being classified as "preferred", these 2 categories were grouped for analysis. Table 1b summarizes the French "what-to-start" guidelines.

cART modification was defined as any change of at least 1 antiretroviral during the first year of treatment. Dosage adjustments and switch to fixed-dose combinations using the same molecules were not considered modifications.

The main reason for treatment modification or interruption was classified at the time of event as virological failure (defined by a confirmed HIV-RNA >200 copies/mL at 6 months or >50 copies/mL at 12 months), toxicity/tolerance issues, simplification of treatment, patient request (including observance issues), and other reasons, including pregnancy and pharmacological interactions.

### Statistical analyses

Qualitative variables were reported as numbers and percentages and were compared with the Chi2 test. Quantitative variables were reported as medians with interquartile range (IQR) and were compared with the Mann-Whitney *U* Test. The Chi2 test for linear trends was used to assess the effect of the year of guideline publication on the outcomes.

Factors associated with cART modifications at 12 months and factors associated with an HIV viral load <50 copies/mL at 12 months were assessed by univariate and multivariate logistic regression modeling. Variations in CD4 counts from baseline were assessed by univariate and multivariate linear regression.

The independent variables were whether the treatments followed the "when-to-start" and "what-to-start" guidelines. The following potential confounders were tested: year guidelines were published, age at cART initiation, risk factors for HIV infection, delay between HIV diagnosis and cART initiation, presence of HBV/HCV coinfection, Centers for Disease Control and Prevention (CDC) stage, HIV viral load, and CD4 count at cART initiation.

After univariate analysis, independent variables and potential confounders were entered in multivariate models if their *P* value was≤ .15; a backward stepwise process was then implemented and models were compared with the Wald test. Baseline CD4 count was forced in the predictive model of CD4 count variation at 12 months. Baseline HIV viral load was forced in the predictive model of undetectable HIV viral load at 12 month. All statistical tests were 2-tailed, and *P* < .05 was considered statistically significant. The viral suppression rate at 12 months was analyzed in an intention to treat (ITT) way (i.e. that all switches were counted as failures). The data were analyzed by Stata software 11.0 (StataCorp LP 2009, College Station, TX). Missing data were avoided by complete case analysis.

## Results

From January 2000 to December 2010, 1365 patients initiated cART accordingly to the inclusion criteria. The demographics and clinical characteristics of the patients at baseline are summarized in Table 2.

**Table 2:** **Demographic and baseline characteristics of patients receiving treatments that did/did not follow the guidelines**

	All population	Followed "when-to-start" guidelines			Followed "what-to-start" guidelines		
Characteristic	N = 1,365	Yes (n = 1,214)	No (n = 151)	P	Yes (n = 1,215)	No (n = 150)	P
Male (%)	69.7	68.6	78.2	0.02	68.7	77.3	0.03
Median Age (years)	37.3	37.5	34.7	0.002	37.1	38.5	-
MSM (%)	41.4	39.0	60.9	<0.001	41.1	44.0	-
HBV/HCV coinfection (%)	13.1	13.3	11.3	-	13.2	12.7	-
CDC stage C (%)	22.6	25.4	0	<0.001	20.2	41.3	<0.001
Median CD4 (/mm^3^)	248	230	513	<0.001	256	182	<0.001
Median HIV-RNA (log cps/mL)	4.8	4.8	4.4	<0.001	4.7	4.9	<0.001
HIV-RNA >10^5^ copies/mL (%)	40.5	41.4	14.3	<0.0001	37.3	48.2	<0.0001

*Abbreviations*: *MSM* men who have sex with men, *HBV* hepatitis B virus, *HCV* hepatitis C virus, *CDC* Centers for Disease Control and Prevention, *HIV* human immunodeficiency virus.

cART not following the current "when-to-start" guidelines was initiated in 151 patients (11%). Treatment not following the guidelines declined from 22% in the 2000-2001 period to 6% in 2010 (*P* < .001). Factors associated with not following the "when-to-start" guidelines at cART initiation included younger age, male gender, men having sex with men, CDC stage A-B, higher CD4 count, and lower HIV viral load at baseline.

One hundred and thirty-two different cART combinations were prescribed; 65 that followed the "what-to-start" guidelines and 67 that were outside the guidelines. These 67 regimens outside the guidelines represented 150 patients and included suboptimal combinations such as 2 or 3 nucleoside/nucleotide reverse transcriptase inhibitors (NRTI) (25 patients), more than 3 drugs (n = 69), 2 NRTI with a fusion inhibitor (n = 10) and other combinations of 3 drugs from 3 different classes (n = 46). cART that followed the current "what-to-start" guidelines was initiated in 1215 patients (89%) and treatments that were outside guidelines were initiated for 150 (11%). Prescription of drugs outside the current guidelines declined from 18% in the 2000-2001 period to 9% in 2010 (*P* < .001). Factors associated with initiating cART that did not follow the "what-to-start" guidelines included older age, male gender, a shorter delay between HIV diagnosis and cART initiation, CDC stage C, lower CD4 count, and higher HIV viral load at baseline. Factors associated with following or not following the guidelines are summarized in Table 2.

Eleven patients died during the first year of follow up. At 12 months, 64.3% of patients had an undetectable HIV-RNA <50 copies/mL, 31.9% had a CD4 count greater than 500/mm^3^, and 43.6% reported at least 1 treatment modification during the first year of treatment. The proportion of patients with HIV-RNA <50 copies/mL at 12 months increased from 46.7% for the 2000-2001 period to 73.1% for 2010 (*P* < .001). Similarly, the proportion of patients with CD4 counts >500/mm^3^ at 12 months increased from 28.6% to 43.2% (*P* < .001). Conversely, the proportion of patients reporting cART modification during the first year decreased from 52.1% to 22.6% (*P* < .001). Reasons for cART modification were virological failure in 6.6% of patients (n = 39), toxicity/tolerance issue in 41.0% (n = 244), simplification in 21.3% (n = 127), patient request in 7.9% (n = 47), and other reasons in 23.2% (n = 138).

The proportion of patients with an HIV viral load <50 copies/mL at 12 months was similar for patients initiating cART with treatments that followed and did not follow the "when-to-start" guidelines, as well as the proportion of patients with a treatment modification during the first year. The median CD4 count was higher at 12 months for patients initiating cART that did not follow the "when-to-start" guidelines (*P* < .001), whereas the CD4 increase from baseline was lower in these patients (*P* = .006).

Conversely, patients initiating cART with treatments that did not follow the "what-to-start" guidelines reported more treatment modifications (*P* < .001), experienced undetectable HIV-RNA less frequently, and had lower CD4 counts and lower CD4 count increases at 12 months than patients initiating cART according to the guidelines (*P* < .001). The 12-month outcome data are summarized in Table 3.

**Table 3:** **Twelve months outcomes after cART initiation according to treatments that did/did not follow the guidelines**

	All population	"when-to-start" guidelines			"what-to-start" guidelines		
Characteristic	N = 1,365	Followed guidelines (n = 1,214)	Outside of guidelines (n = 151)	P	Followed guidelines (n = 1,215)	Outside of guidelines (n = 150)	P
cART modification (%)	43.6	43.7	43.4	-	41.2	62.7	<0.001
12 months CD4 (/mm^3^) (median [IQR])	395	369	619	<0.001	406	313	<0.001
	[241-538]	[224-506]	[504-766]		[257-548]	[148-484]	
CD4 gain (/mm^3^) (median [IQR])	137	139	89	0.006	144	69	<0.001
	[33-238]	[39-239]	[0-216]		[40-239]	[0-204]	
12 months HIV-RNA (log copies/mL) (median [IQR])	1.7	1.7	1.7	-	1.7	1.9	<0.001
	[1.6-2.3]	[1.6-2.3]	[1.7-2.4]		[1.6-2.2]	[1.7-3.4]	
HIV-RNA <50 copies/mL (%)	64.6	64.3	66.9	-	66.8	47.3	<0.001
Deceased (%)	0.8	0.9	0	-	0.6	2.7	0.007

*Abbreviations*: *IQR* interquartile range, *cART* combined antiretroviral therapy, *HIV* human immunodeficiency virus.

After univariate regression analysis, treatments not following the "when-to-start" guidelines were associated with decreased CD4 counts (crude Regression Coefficient [cRC] = -39.5, 95% CI -66.4; -12.6, *P* = .004), but was not associated with treatment modification (crude odds ratio [OR] = 0.95, 95% CI 0.67-1.33, *P* = .75) or HIV-RNA <50 copies/mL at 12 months (cOR = 1.12, 95% CI 0.78-1.60, *P* = .54). Not following the "what-to-start" guidelines was associated with a higher treatment modification rate (cOR = 2.39, 95% CI 1.69-3.94, *P* < .001), decreased rate of HIV-RNA <50 copies/mL (cOR = 0.45, 95% CI 0.32-0.63, *P* < .001), and decreased CD4 count at 12 months (cRC = -36.0, 95% CI -63.0; -9.0, *P* = .009).

Factors independently associated with cART modification during the first year of treatment were female gender, CDC stage C, year of guideline publication (with a tendency toward less treatment modification over time), and a treatment that did not follow the "what-to-start" guidelines. A sensitivity analysis was performed by excluding simplification of treatment from other causes of cART modification. This analysis gave similar results (data not shown).

Factors independently associated with a greater increase in CD4 counts at 12 months were CDC stage A-B, baseline HIV-RNA <105 copies/mL, absence of HBV/HCV coinfection, pursuit of the same cART during the first year, and initiation of cART after 2004. Treatments that followed the "what-to-start" guidelines were not independently associated with CD4 count variation at 12 months.

Factors independently associated with an undetectable HIV-RNA at 12 months were CDC stage A-B, pursuit of the same cART during the first year, initiation of cART after 2006, and treatments following the "what-to-start" guidelines.

Treatments that followed the "when-to-start" guidelines were not independently associated with cART modification, CD4 increase, or undetectable HIV-RNA at 12 months. The results of these different models are summarized in Table 4.

**Table 4:** **Factors associated with 12-month outcomes following cART initiation**

	cART modification before 12 month		HIV-RNA <50 copies/mL at 12 month		CD4 variation from baseline	
Characteristic	aOR*	*P*	aOR*	*P*	aRC*	*P*
cART modification	**	**	0.19 (0.15-0.24)	<.001	-68.2 (-84.5;-52.0)	<.001
Outside "what-to-start" guidelines	1.90 (1.45-2.49)	<.001	0.67 (0.45-1.00)	.05	-	-
Year of guidelines (ref: 2000)						
2002	0.63 (0.42-0.97)	.03	0.96 (0.60-1.53)	-	4.4 (-26.2-34.9)	-
2004	0.57 (0.37-0.87)	.009	1.44 (0.90-2.31)	-	(14.0-75.5)	.004
2006	0.33 (0.22-0.58)	<.001	2.07 (1.31-3.28)	.002	51.4 (22.2-80.6)	.001
2008	0.40 (0.27-0.58)	<.001	2.05 (1.32-3.17)	.001	35.9 (7.7-64.0)	.01
2010	0.40 (0.26-0.62)	<.001	1.95 (1.19-3.20)	.008	27.6 (-3.9-59.1)	-
Male gender	0.76 (0.59-0.96)	.02	-	-	-	-
HBV/HCV coinfection	-	-	-	-	-26.1 (-49.6;-2.6)	.03
CDC stage C	1.90 (1.45-2.49)	<.001	0.54 (0.40-0.73)	<.001	-39.4 (-60.5;-18.3)	<.001
HIV-RNA >10^5^ cps/mL	-	-	**	**	61.0 (44.3-77.7)	<.001

*Abbreviations*: *HBV* hepatitis B virus, *HCV* hepatitis C virus, *CDC* Centers for Disease Control and Prevention, *HIV* human immunodeficiency virus, *aOR* adjusted odds ratio, *aRC* adjusted regression coefficient.

Variables not retained in the final multivariate models: age, HIV risk factor, baseline CD4, delay from HIV diagnosis to cART initiation, adherence to "when-to-start" guidelines.

*Adjusted on the other covariates.

**Covariate not in the model.

## Discussion

The objective of this study was to identify the relationship in daily practice between following treatment guidelines and the outcome within 1 year of initiating cART. Following the guidelines appeared overall satisfactory in this well-defined population of cART-naïve HIV-infected adults. However, 11% of patients were treated outside of the "when-to-start" guidelines and 11% outside of the "what-to-start" guidelines. Similar results have been observed in other European and Australian studies [9],[12]-[14]. A higher rate of treatments initiated outside the guidelines was reported in 2 studies from the United States (not following the "what-to-start" guidelines in 46%, not following the "what-to-start" guidelines in 21-56%) [10],[11], but one of these studies included patients treated prior to the cART era in 1996, which could explain such differences.

The reasons for initiating treatment outside the guidelines appeared remarkably different when considering whether or not the "when-to-start" or "what-to-start" guidelines were followed. Patients initiating cART outside the "when-to-start" guidelines were typically young men having sex with men, less immunocompromised than the other patients, who initiate cART at a higher CD4 count level than those recommended at that time. Since the recommended CD4 count threshold has increased over time, patients starting cART above this threshold generally anticipated the following guidelines. Initiating treatment above the given CD4 count threshold was also observed in Danish and Australian studies [13],[15], with a similar tendency for the rate to decline over time in the former study. As a consequence of the evolution of guidelines, the practice of not following the "when-to-start" guidelines declined over time and should be minimal in the future since the recently published French guidelines, like the American guidelines [23], now recommend initiating cART in every patient irrespective of CD4 count [7].

The 1-year outcomes were quite similar whether or not the "when-to-start" guidelines were followed. Not surprisingly, patients initiating treatment outside these guidelines had higher 12 month CD4 counts but lower CD4 increases than other patients. The virological efficacy was, however, identical between groups, and there was a similar rate of treatment modification during the first year of treatment. Therefore, neither a beneficial nor a detrimental evolution was observed after 1 year of follow-up in patients initiating treatment outside the "when-to-start" guidelines, whereas long-term differences could not be ruled-out in this study. Additionally, initiating cART earlier in these patients could have been associated with other benefits not measured in this study, such as a reduction in infectiosity and contagiousness.

Following the "when-to-start" guidelines was better during the studied period than during the pre-cART (<1996) and early-cART eras (1996-1999), when the rapid evolution of knowledge led physicians to largely anticipate the guidelines [24]. Similar to "when-to-start" guidelines, prescribing treatments outside of the "what-to-start" guidelines declined over time, whilst the reasons for initiating cART outside of these guidelines appeared to be different. Patients who initiated treatment outside these guidelines appeared to be more immunocompromised than others, whereas demographic factors were not as discriminant. Despite the fact that HBV/HCV coinfections may require specific considerations for cART, this characteristic was not associated with use of non-recommended treatments. Reasons for initiating cART that were outside of the "what-to-start" guidelines differ markedly in the literature. Intravenous drug usage [13], female gender [14], a higher education level [14], age younger than 60 years [10], white race [10], city or state of treatment [10],[15], year of treatment [10],[11],[14],[15], lowest [10] or highest [10],[14] CD4 count, and lowest [10],[11],[14] or highest [10],[12],[14] HIV viral load have all been reported as associated with a non-recommended treatment, precluding the identification of a unique pattern.

Twelve-month outcomes were remarkably different regarding whether or not "what-to-start" guidelines were followed. Patients initiating cART outside of these guidelines had significantly worse immunological and virological outcomes and more treatment modifications during the first year than patients treated within these guidelines. A similar result was observed in the Swiss Cohort [14] in which an HIV viral load <400 copies/mL at 1 year was significantly less frequent when cART was initiated outside the IAS-USA guidelines. However, this result was no longer significant when considering a <50 copies/mL threshold, and there was no difference in CD4 count increases, unlike in our study. Differences in demographic characteristics may explain such differences, since patients in the Swiss Cohort were slightly younger, more frequently intravenous drug users, and more frequently coinfected with HCV than our patients. Similarly, more patients initiated cART with CD4 counts below 200/mm^3^ in this study, whereas more patients initiated cART with CD4 counts between 200 and 350/mm^3^ in our study. However, the distribution of HIV viral load at initiation of treatment was similar between the 2 cohorts.

Treatments outside of the "when-to-start" guidelines were not associated with virological or immunological outcome at 12 months. Conversely, complex interactions were observed between treatment modifications during the first year, immunological and virological response at 1 year, and following the "what-to-start" guidelines. CDC stage C and the year of guideline publication were common predictors of all 12-month outcomes. Similarly, cART modification was negatively associated with an undetectable HIV-RNA at 12 months and with CD4 count increases from baseline. Female gender was associated with more treatment modifications during the first year, but was not associated with immunological and virological response. As previously reported [25],[26], HBV/HCV coinfection was associated with lower CD4 count increases from baseline, but was not associated with more treatment modifications or worse virological response. Higher HIV viral load was associated with a greater CD4 count increase, which has also been previously reported [14],[27]. Finally, not following the "what-to-start" guidelines was independently associated with a worse virological response. Contrary to the Swiss Cohort study [14], age, HIV risk factors, and baseline CD4 counts were not associated with 12-month outcomes. Differences in populations and the selection of variables can explain such discrepancies between the study results. However, our study is the first to report a clear association between following the "what-to-start" guidelines and the virological response at 1 year using the universally accepted cut-off of <50 copies/mL, irrespective of the degree of immunodeficiency.

Limitations of this study include the follow-up that was voluntarily limited to 1 year in order to assess the effect of the first line of cART. Data on adherence and compliance were not collected, as well as associated comorbidities, which could have influenced the timing of cART initiation or the choice of drugs. A center effect could not be excluded, but the monocentric nature of the study allowed us to better characterize the predictors of outcome, since the study was less susceptible to heterogeneity in patient care than multicentric ones. Since the French guidelines for treating HIV infection are widely recognized in France, we referred to these guidelines rather than those of the IAS-USA or other international organizations, but we speculate that given the convergence of these guidelines over time, our conclusions would have been the same using a different set of guidelines. Excluding acute infection from the study could be another possible limitation since many physicians have been treating acute infection far before guidelines indicated to do so. However, since recommendations on the treatment of primary infection evolved with time, excluding acute infections provided homogeneity in our study population.

## Conclusions

In conclusion, initiating treatments that did not follow the "when-to-start" guidelines was related to cART initiation at higher CD4 counts than the recommended level at the time, mainly in young men who have sex with men, and was not associated with a significantly different 12-month outcome than observed in the same population during that period of time. Conversely, not following the "what-to-start" guidelines was not observed in any specific population and led to significantly worse virological outcomes at 12 months and more treatment modifications during the first year of cART. Efforts should be made to promote adherence to the "what-to-start" guidelines and to assess the adequacy of prescriptions within these guidelines.

## Authors' contribution

LC designed the study, collected the data, participated to data analysis and interpretation, drafted and revised the article. TB analyzed the data, participated to data interpretation and revised the article. PV designed the study, analyzed the data participated to data interpretation and revised the article. CB collected the data, participated to data interpretation and revised the article. TP collected the data, participated to data interpretation and revised the article. TF collected the data, participated to data interpretation and revised the article. CC designed the study, collected the data, participated to data analysis and interpretation and revised the article. All authors read and approved the final manuscript.

## Abbreviations

aOR: Adjusted odds ratio
aRC: Adjusted regression coefficient
CDC: Centers for disease control and prevention
cART: Combined antiretroviral therapy
HBV: Hepatitis B virus
HCV: Hepatitis C virus
HIV: Human immunodeficiency virus
IQR: Interquartile range
NRTI: Nucleoside/nucleotide reverse transcriptase inhibitor
